# Single-Visit Endodontic Management of a Complex Endo-Periodontal Lesion in an Oncologic Patient with Systemic Comorbidities: A 12-Month Case Report

**DOI:** 10.3390/dj13090388

**Published:** 2025-08-26

**Authors:** Salvatore Distefano, Francesco Bellucci, Salvatore La Rosa, Giuseppe Evola, Carmelo Federico, Giovanni Barbagallo, Roberto Sammarco, Pietro Valerio Foti

**Affiliations:** 1 Centro Odontoiatrico Distefano s.r.l., 95128 Catania, Italy; 2 Private Practice, 83100 Avellino, Italy; 3 Private Practice, 95100 Catania, Italy; 4 General and Emergency Surgery Department, Garibaldi Hospital, Piazza Santa Maria di Gesù 5, 95100 Catania, Italy; 5 Private Practice, 95123 Catania, Italy; 6 Italian Academy of Microscopic Dentistry (AIOM), Via Andrea Anfossi 57, 92010 Lampedusa, Italy; 7 Department of Medical Surgical Sciences and Advanced Technologies “G.F. Ingrassia”, University of Catania, 95123 Catania, Italy; pietrofoti@hotmail.com; 8 Radiology I Unit, University Hospital Policlinico “G. Rodolico-San Marco”, Via Santa Sofia 78, 95123 Catania, Italy

**Keywords:** root canal instrumentation, root canal treatment, CBCT in endodontics, low-dose CBCT in endodontics, endodontics and systemic pathologies, microscope in endodontics, bioceramics, external cervical root resorption, endodontic-periodontal lesion

## Abstract

**Background:** The endodontic management of patients with complex systemic conditions represents a significant clinical challenge, particularly in cases involving combined endodontic-periodontal lesions and extensive root resorptions. This case report, accompanied by a recent and targeted literature analysis, describes the single-session treatment of an oncologic patient with multiple systemic comorbidities, presenting with a communicating periapical and endodontic-periodontal lesion (EPL), as well as external cervical root resorption (ECR). **Methods:** Root canal treatment (RCT), combined with ECR repair, was performed using bioceramic materials, via thorough management and planning with cone-beam computed tomography (CBCT) and the aid of an operating microscope. **Results:** At the 12-month follow-up, complete clinical and radiographic healing was observed, including regeneration of the periodontal ligament and full functional recovery of the tooth. **Conclusions:** Contrary to what has been reported in the literature, where several studies highlight lower healing rates in patients with systemic comorbidities undergoing endodontic treatment, the present case report demonstrates a favorable and stable outcome. Moreover, the possibility of concentrating the intervention into a single session represents an additional advantage for this category of patients, who often face limited physical and temporal resources due to their concurrent oncologic condition.

## 1. Introduction

Several studies have demonstrated that compromised systemic conditions and states of systemic hyperinflammation may be associated with reduced healing rates following root canal treatment (RCT) [1,2,3,4]. However, few studies in the literature have included a sufficiently large cohort of patients with systemic and oncologic comorbidities in order to investigate the effects and efficacy of endodontic therapy in the treatment and resolution of endodontic lesions [1,2,3,4].

Moreover, the presence of systemic impairments, myelosuppression, and systemic hyperinflammatory states, as well as elevated levels of C-reactive protein (CRP) and high-sensitivity C-reactive protein (hs-CRP), can negatively impact healing outcomes after RCT [2,3,5].

These acute-phase proteins not only tend to an increase in response to endodontic lesions, but also serve as markers of both systemic and local inflammation, often associated with chemical mediators such as interleukin-6 (IL-6), interleukin-1 beta (IL-1β) tumor necrosis factor (TNF), and reduced expression of angiogenic and vascular factors. These elements collectively contribute to a diminished response to RCT, and may promote and sustain the development of periapical lesions [2,3,4,5,6,7].

With regard to CRP, research suggests that it may serve as a reliable biomarker of the intensity and progression of odontogenic infections. CRP has demonstrated greater sensitivity than white blood cell count, and could become a valuable marker for integration into prognostic models aimed at assessing both the presence of infection and the effectiveness of dental treatment [8]. In particular, hs-CRP is a useful hematochemical biomarker, capable of detecting even low-grade chronic inflammation, and it can also be used to integrate the assessment of cardiovascular risk profile [3,5].

The recovery of a severely compromised tooth often requires comprehensive biological assessments and multidisciplinary operative planning. Within the scope of root canal therapy, the close anatomical relationship between the endodontic and periodontal compartments necessitates accurate diagnosis of the underlying pathological factors in order to establish appropriate and effective treatment strategies [9,10,11].

In endodontics, the literature reports numerous studies on the use of calcium-silicate-based cements for root canal obturation, root structure repair, and the maintenance and management of pulp vitality [12,13].

Moreover, a recent systematic review confirms and highlights the superior antibacterial properties of Biodentine^®^ (Septodont, Saint-Maur-des-Fossés, France) when compared to other root canal filling and repair materials. In particular, it has shown notable efficacy against *Enterococcus faecalis* [14], a Gram-positive facultative anaerobic coccus known for its resistance to endodontic treatment and its association with persistent endodontic infections [14].

These cements offer the advantage, in certain clinical scenarios, of optimizing procedural workflows within a single session [12]. Unlike first-generation bioceramic materials such as Mineral Trioxide Aggregate (MTA), newer bioceramics demonstrate reliable setting and hardening properties, even in the presence of inflamed tissues, thus eliminating the need for intermediate calcium hydroxide dressings. In addition, they offer higher compressive strength and superior color stability in treated teeth compared to MTA—advantages that are particularly relevant for the treatment and structural repair of anterior teeth [12,15,16].

Regarding the effects of bioceramic cements in direct contact with the periodontal ligament, in vivo evidence remains limited [15]. Several studies have investigated these effects on human periodontal ligament stem cells (hPDLSCs) using in vitro cytotoxicity and immunological assays [15,17], as well as in animal models [18].

Bioceramic cements can also be used in the management of root resorption [19], a complex condition that may present with varying degrees of severity and destruction of the dental root. Root resorptions are mediated by odontoclastic activity, typically triggered by bacterial, inflammatory, or traumatic insults, and are generally classified as invasive internal root resorption (IRR), invasive external root resorption (IER), and external cervical resorption (ECR) [19].

The progression and extent of tissue destruction are closely correlated with the duration of inflammatory stimuli [20]. In the absence of necrosis accompanied by liquefaction or abscess formation, these lesions are often asymptomatic. Clinical signs may include crown discoloration, resulting from bacterial colonization and tissue degradation [19,20].

A classification of ECR based on cone-beam computed tomography (CBCT) imaging has been proposed in the literature, as described by Patel et al. (2018) [21] and later adopted by the European Society of Endodontology (ESE). This classification is structured around three main parameters: the lesion’s height, its circumferential extension, and its proximity to the root canal.

The position of the alveolar bone crest is best visualized in the sagittal sections of CBCT images. The vertical extent of the ECR lesion is classified into four categories:

1. Supracrestal: The lesion is located at or coronal to the cemento–enamel junction (CEJ), above the alveolar bone crest;
2. Subcrestal: The lesion extends into the coronal third of the root, apical to the alveolar crest;
3. Middle third of the root involved;
4. Apical third of the root involved.

The circumferential extension of the lesion is assessed based on its maximum spread around the root’s surface, which is more accurately visualized in the axial CBCT sections, and is classified as follows:

- A: ≤90°;
- B: >90° and ≤180°;
- C: >180° and ≤270°;
- D: >270°.

The proximity to the root canal is also evaluated using axial CBCT images and is classified as follows:

- d (dentin): Lesion confined to dentin;
- p (pulpal): Likely pulpal involvement.

It provides valuable diagnostic and therapeutic support in the treatment planning of cases involving external cervical resorption.

Radiological diagnosis is therefore essential for approaching such lesions: intraoral radiography can reveal the presence of resorption, but does not allow for the accurate assessment of its boundaries and extent due to the limitations of two-dimensional imaging, such as distortion, superimposition of anatomical structures in a single plane, and beam attenuation caused by tissue thickness [22]. CBCT is considered the gold standard for correctly identifying the lesion within the dental structure and for determining its extent in the periradicular tissues [21,22]. These are critical elements in formulating an appropriate treatment plan and ensuring precision during the operative procedures aimed at salvaging the affected tooth.

In cases of severely compromised teeth with extensive root resorption, communications may occur between the endodontic and periodontal compartments, resulting in an endo-periodontal lesion (EPL). Bacterial and inflammatory irritants originating from the pulp chamber can invade and damage the periodontal ligament tissues, as observed in the case presented.

This article describes the in vivo single-visit management and positive endodontic and periodontal outcomes of treating a case of root resorption in the right maxillary first premolar (tooth 1.4) using calcium-silicate-based materials in sealer and putty formulations on a patient with significant systemic comorbidities. Several studies have reported greater challenges and reduced healing potential following RCT in similar cases [15], but there is no unanimous consensus regarding the treatment and long-term preservation of severely compromised teeth [17].

At the 12-month follow-up, the tooth exhibited clear radiographic healing, along with the resolution of clinical signs and symptoms.

## 2. Case Report

The patient was a 68-year-old female with a medical history significant for smoking approximately 15 cigarettes per day, type 2 diabetes mellitus managed with oral hypoglycemic agents, arterial hypertension, and cardiovascular disease (CVD) with moderate mitral valve insufficiency.

Remote medical history included a breast carcinoma treated with surgery and chemo-/radiotherapy approximately 10 years before.

The patient is affected by vasomotor rhinitis and is currently diagnosed with stage III endometrial carcinoma with extrauterine extension of the neoplastic mass. Recent clinical and laboratory records reveal high values of glycated hemoglobin (HbA1c) (7.4%, while the reference range is between 5.6% and 6.5%), leukopenia (with a white blood cell count of 2500/µL, significantly below the normal reference range of 4000–10,000/µL) and anemia, indicative of myelosuppression.

Moreover, the patient presented with a hs-CRP level of 11.97 mg/L, indicating a condition of extensive systemic inflammation.

According to reference ranges, values are interpreted as follows:

- Negative: <0.50 mg/L;
- Non-acute inflammatory process: 0.50–1.00 mg/L;
- Mild acute inflammation: 1.00–10.00 mg/L;
- Extensive inflammation: >10.00 mg/L.

Although hs-CRP has low specificity, its high sensitivity in detecting systemic inflammatory conditions is well established. Alterations in this parameter, particularly when combined with other comorbidities, may negatively impact the healing process of dental and endodontic treatments.

### 2.1. Current Medical Therapy

The patient’s medical history revealed the regular intake of multiple systemic medications. The prescribed pharmacological regimen included the following: Pantoprazole 20 mg once daily for gastric protection; Acetylsalicylic acid 100 mg daily as antiplatelet therapy; Carvedilol 6.25 mg twice daily for cardiovascular management; Metformin 800 mg twice daily for glycemic control in type 2 diabetes; and Ezetimibe 10 mg daily for dyslipidemia. Additionally, the patient was undergoing oncological treatment with Carboplatin and Paclitaxel, administered according to the oncologist’s protocol. The patient reported a previous treatment with alendronate approximately four years before, and in the last two years she was also receiving Denosumab therapy under medical indication for the management of cancer-related bone disease. This complex systemic condition was taken into careful consideration during treatment planning.

She was undergoing antibiotic therapy for two days prior to evaluation, consisting of amoxicillin with clavulanic acid administered every twelve hours, as prescribed by her primary care physician.

### 2.2. Clinical Examination

**Figure 1:**
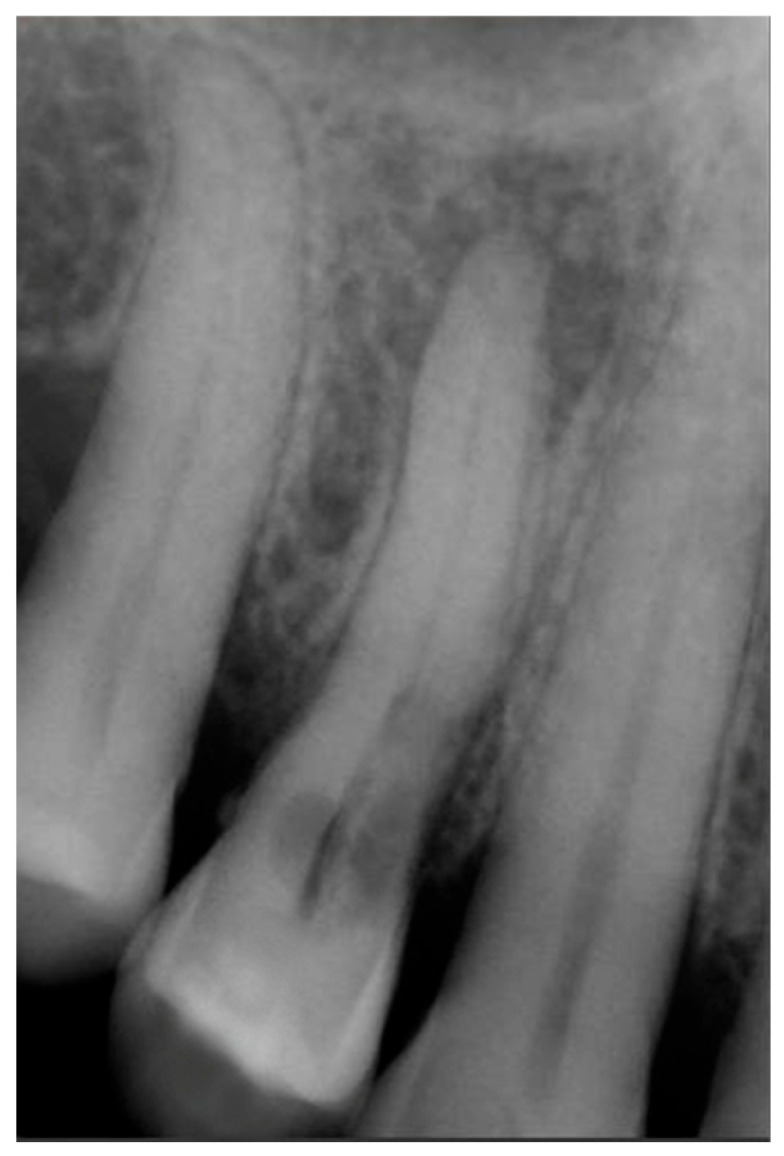
Intraoral radiography of tooth 1.4 showing root resorption, LP and EPL with mesiocervical widening of the lamina dura space.

Signs of parafunctional habits and bruxism were noted, along with compromised periodontal status and poor oral hygiene. Multiple carious lesions were present, and the patient exhibited a gag reflex during examination. The patient presented complaining of spontaneous pain and pain upon mastication in the right maxillary region. Tooth 1.4 showed significant pain on percussion and a pathological periodontal probing depth at the mesiolingual site. Intraoral radiography revealed an extensive cervical root resorption zone and a periapical lesion (PL) (Figure 1).

**Figure 2:**
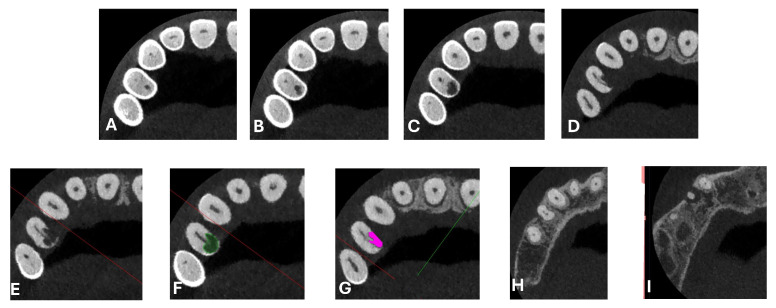
Axial CBCT images of tooth 1.4, showing its ECR and PL. (**A**–**E**) Sequential scrolling on the axial plane from the cervical area to the middle third of the root; (**F**,**G**) Segmentation of Figure 2E using the advanced software functions iRYS^®^ (MyRay, Imola, Italy); (**H**,**I**) Sequential scrolling on the axial plane of the apical third of the root, including the PL.

**Figure 3:**
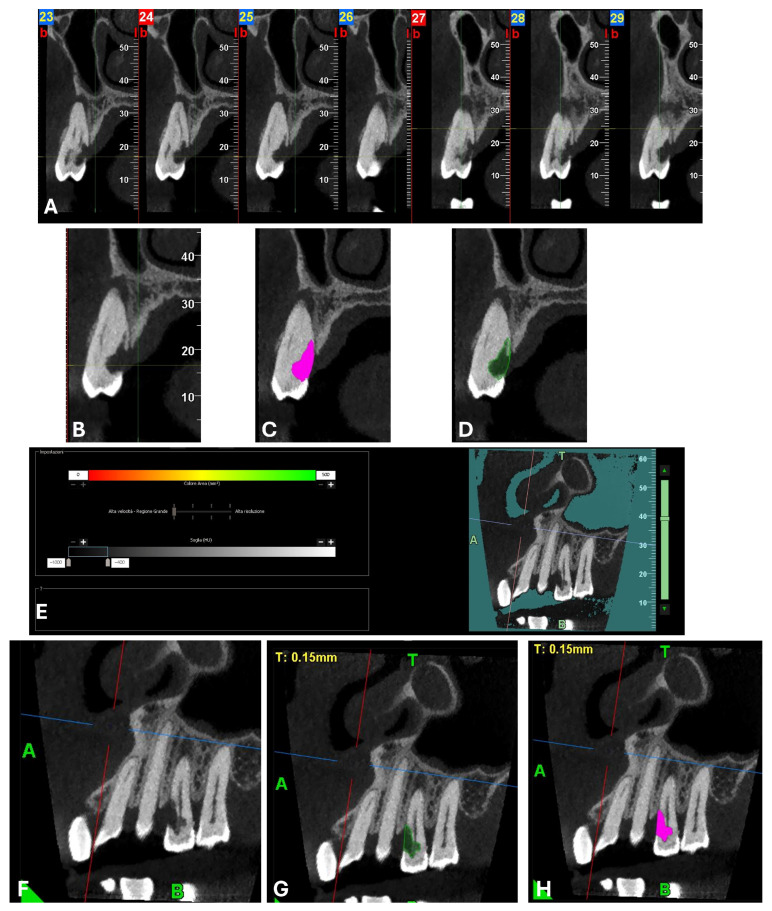
Cross-section and Pano-rex CBCT images of tooth 1.4 and its ECR and EPL. (**A**,**B**) Sequential sagittal scrolling allows the evaluation of the lesion in the bucco-lingual direction and its continuity along the root axis; (**C**,**D**) Segmentation of Figure 3B using the advanced software functions iRYS^®^ (MyRay, Imola, Italy); (**E**) Interface of the aforementioned software to obtain segmentation on Pano-rex image. (**F**) Pano-rex image of tooth 1.4 and its EPL. (**G**,**H**) Segmentation of Figure 3F using the advanced software functions. The colored lines crossing the images represent the orthogonal planes (axial, coronal, and sagittal) in the multiplanar reconstruction. Their correct use and orientation allow for an accurate identification of the extent of the lesions in all spatial planes and a detailed analysis of the endodontic anatomy. The green letters are the coordinates of the image: A: anterior, B: bottom, T: Top.

**Figure 4:**
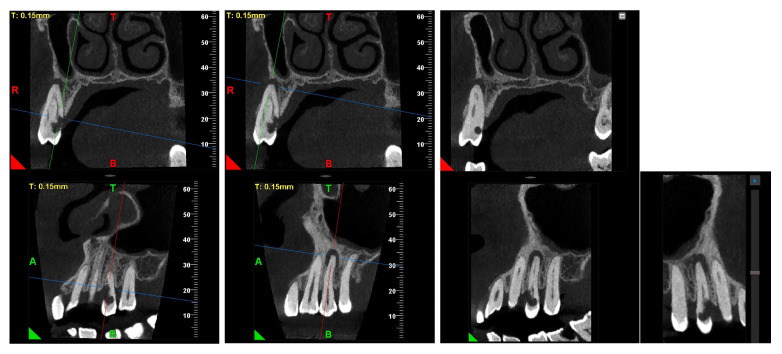
Pano-rex and cross-sectional CBCT images of tooth 1.4, showing its ECR, EPL, and PL. The colored lines crossing the images represent the orthogonal planes (axial, coronal, and sagittal) in the multiplanar reconstruction. Their correct use and orientation allow for an accurate identification of the extent of the lesions, including possible relationships with the maxillary sinus, in all spatial planes and a detailed analysis of the endodontic anatomy. The green and red letters are the coordinates of the image: A: anterior, R: right, B: bottom, T: Top.

**Figure 5:**
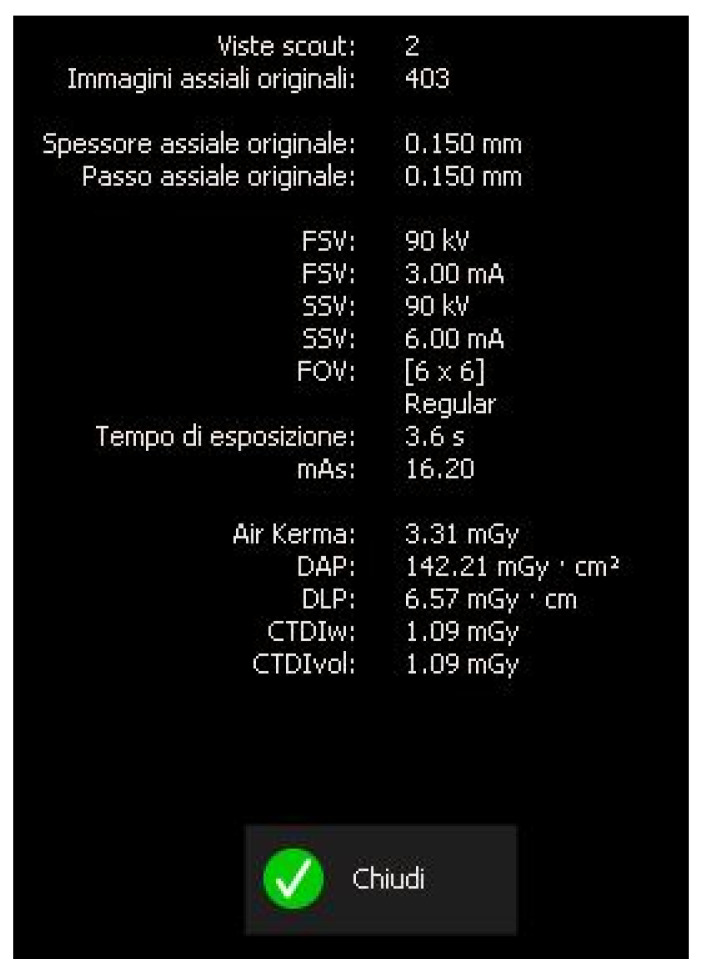
Exposure parameters of CBCT image.

A CBCT examination (X9 Pro^®^, MyRay, Imola, Italy), performed with dedicated field of view (FOV) (6 × 6) and exposure parameters, was essential for accurate diagnosis and treatment planning. This device optionally features a specific Booster function for users with advanced expertise, which allows for further customization of exposure parameters to optimize the examination more effectively. Optimal management of the FOV, properly centered and limited to the anatomical area of interest, in this specific case slightly extended (6 × 6) to clearly rule out a possible involvement of the maxillary sinus in agreement with the attending otorhinolaryngologist, enabled the acquisition of broader and more detailed clinical and diagnostic information, which is essential in endodontics. Moreover, it allowed for a significant reduction in radiation dose while optimizing spatial resolution [22]. The imaging clarified the extent of the resorption and defined its perimeter, demonstrating an endodontic-periodontal communication, classified as 3Bp according to Patel’s classification [21] (Figure 2, Figure 3, Figure 4 and Figure 5).

### 2.3. Clinical Management

In agreement with the patient and accompanying family members, and after consultation with the treating oncologist, the least invasive treatment approach was chosen. After a thorough scaling procedure aimed at improving oral hygiene, the procedure was initiated under plexus anesthesia and with isolation of the operative field, taking care to minimize trauma when manipulating tissues with the hook.

**Figure 6:**
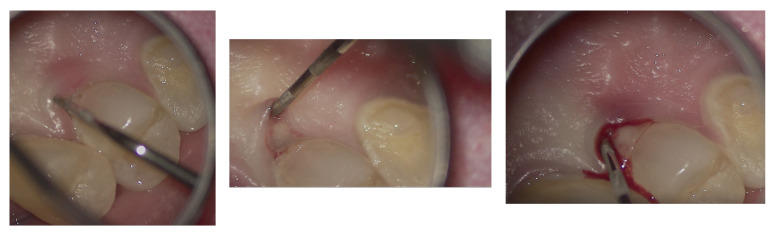
Microscopic exploration and probing of EPL under the operating microscope.

**Figure 7:**
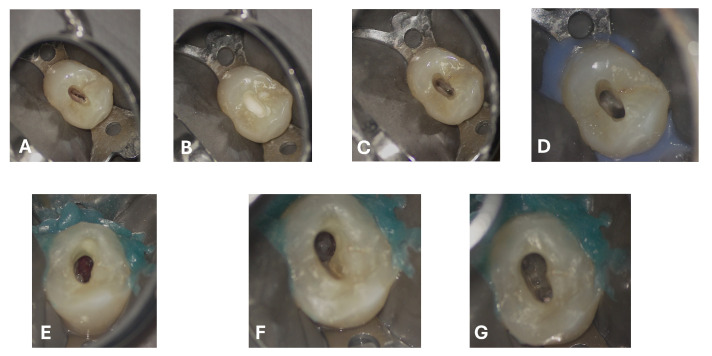
Access cavity prepared under microscopic vision, highlighting the balance between preservation of dental tissue and achievement of adequate access to the root canal system and the lesion. (**A**) Opening and refinement of the cavity and access to the root canal system; (**B**–**D**) Irrigation phases with visible effervescence. Following the progressive definition and assessment of the extent of the access cavity to the resorptive lesion, the following can be observed: (**E**) Presence of inflammatory tissue within the cavity; (**F**,**G**) Debridement of the inflammatory tissue associated with root resorption; chamber and root canal system cleaned and sealed.

The entire procedure was performed under an operating microscope, Zumax 2380^®^, with an integrated HP camera (Zumax Medical Co., Ltd., Suzhou, China). In this case, the endodontic access cavity was prepared using a minimally invasive approach, aiming to preserve as much dental tissue as possible while ensuring adequate control and access to both the root canals and the cervical resorption lesion (Figure 6 and Figure 7).

Scouting was performed manually using a K-file #10 (FKG Dentaire, La Chaux-de-Fonds, Switzerland), and the working length was determined with an electronic apex locator (Root ZX Mini, J. Morita Corporation, Tokyo, Japan). The canals were fully treated using mechanical nickel–titanium rotary instruments—initially with FKG EVO files, followed by the use of FKG XP instruments (FKG Dentaire, La Chaux-de-Fonds, Switzerland). The XP-endo^®^ Shaper 30.04 was employed to maximize circumferential contact with the root canal walls while preserving their original anatomy (generally described as a 4% taper instrument, but characterized by a nickel-titanium (NiTi) alloy and an adaptive design that allows it to expand from an initial 1% taper).

A sequential irrigation protocol was implemented with the alternating use of sodium hypochlorite (NaOCL) and ethylenediaminetetraacetic acid (EDTA) [23]. To ensure that the two irrigants acted effectively and synergistically without interaction, distilled water was used as an intermediate rinse. Specifically, the irrigation protocol included:

- 5% NaOCl (Niclor^®^ 5%, Ogna Laboratori Farmaceutici, Muggiò, Italy): delivered using a 30 G side-vented needle (NaviTip^®^, Ultradent Products, Inc., South Jordan, UT, USA) and a 10 mL syringe (5 mL per canal), 2 mm short of the working length, at a flow rate of 1 mL per 10 s. The procedure was repeated four times per canal.
- Distilled water (Eurospital S.p.A., Trieste, Italy): delivered using a 30 G side-vented needle (NaviTip^®^, Ultradent Products, Inc., South Jordan, UT, USA), 5 mL per canal, used between the irrigants.
- 10% EDTA (Tubiclean^®^, Ogna Laboratori Farmaceutici, Muggiò, Italy): delivered using a 30 G side-vented needle (NaviTip^®^, Ultradent Products, Inc., South Jordan, UT, USA), 1 mL per canal, with each application followed by 20 s of activation, repeated three times.

All irrigants were activated using passive ultrasonic irrigation (PUI) with ultrasonic tips, and the final stages of irrigation were performed with XP-endo^®^ Finisher instruments (FKG Dentaire, La Chaux-de-Fonds, Switzerland) to enhance cleaning efficiency and ensure the optimal penetration of the irrigants within the root canal system [24]. Finally, the final irrigation was performed with EDTA followed by rinse with distilled water.

After drying and electronic confirmation of the working length, the canals were obturated with gutta-percha cones and the bioceramic sealer BioRoot™ RCS (Septodont, Saint-Maur-des-Fossés, France).

**Figure 8:**
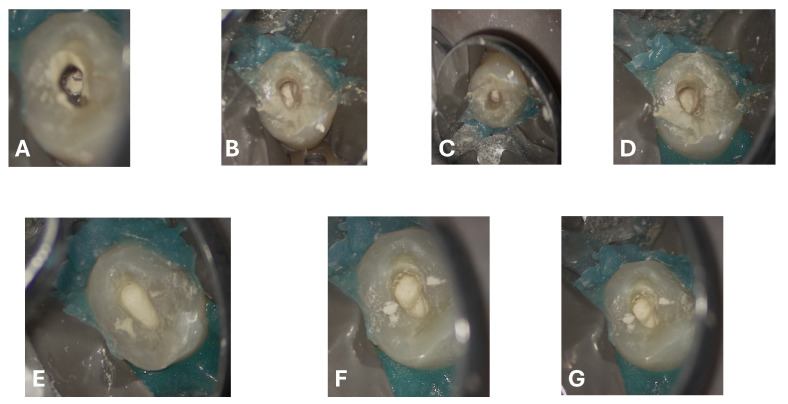
Definition and visual inspection of the root resorption area under the operating microscope, followed by obturation under magnification. (**A**–**G**) Sequential progression of bioceramic (Biodentine) deposition and condensation.

Regarding the ECR lesion, after thorough debridement of the granulation tissue (Figure 7F,G) and after achieving hemostasis, the subcrestal defect was filled and restored with Biodentine^®^ (Septodont, Saint-Maur-des-Fossés, France) in a putty formulation (Figure 8), delivered into the cavity using the MAP System^®^ (Micro-Apical Placement System, Produits Dentaires SA, Vevey, Switzerland), which allows precise placement and controlled condensation under magnification (Supplementary Video S1). This choice was guided by the well-documented properties of calcium-silicate-based cements to promote cementum deposition and the adhesion of a new periodontal ligament.

**Figure 9:**
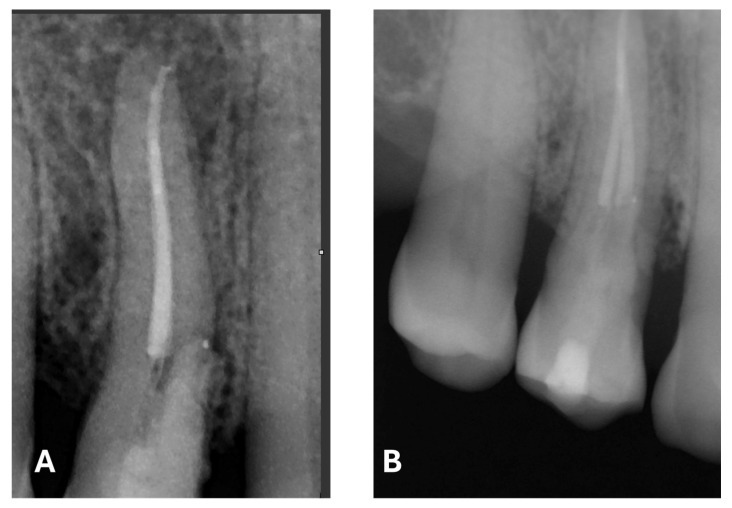
Post-treatment radiograph with composite filling. (**A**) focus on the periapical lesion, root canal obturation and Biodentine application. (**B**) focus on composite filling. Note the radiographic evidence of widening of the lamina dura in the mesiocervical region.

After 15 min from the application of Biodentine, and once the stability of the setting was verified, tooth 1.4 was restored with a composite filling (Figure 9). The procedure, performed under rubber dam isolation in a single operative session, began after re-cleaning the enamel–dentin margins with selective etching using 35% silicon-free phosphoric acid [Ultra-Etch^®^, Ultradent Products Inc., South Jordan, UT, USA], applied for 30 s on enamel and 15 s on dentin. The surface was then thoroughly rinsed and gently air-dried. A primer and bonding adhesive [Prime & Bond Active^®^, Dentsply Sirona, Konstanz, Germany] were applied and light-cured for 20 s with an LED curing unit [VALO Grand^®^, Ultradent Products Inc., South Jordan, UT, USA]. Finally, the composite material [Omnichroma^®^, Tokuyama Dental Corp., Tokyo, Japan] was placed using the incremental layering technique and polymerized for 20 s.

The radiographic quality of endodontic treatment—particularly in regard to canal filling density, appropriate taper, and respect for the working length—is significantly associated with a lower incidence of endodontic failure. Inadequate fillings, overextensions, underfillings, or the presence of voids within the canal have been correlated with a higher likelihood of persistent periapical lesions and a reduced treatment success rate [25].

### 2.4. Follow-Up

**Figure 10:**
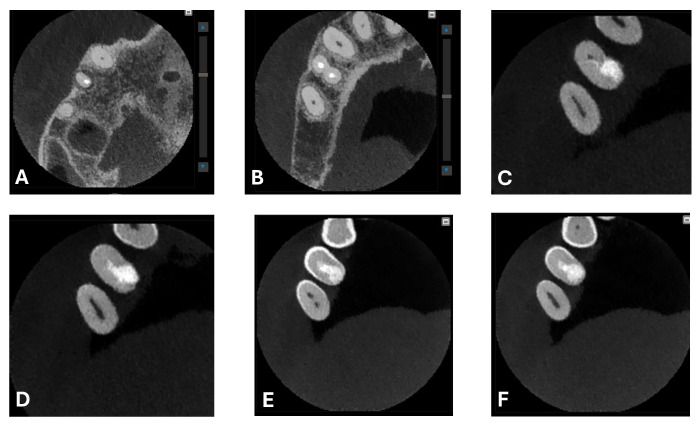
Axial CBCT images of tooth 1.4 after six months of healing. (**A**–**F**) Sequential scrolling of the root of tooth 1.4 after six months of healing.

**Figure 11:**
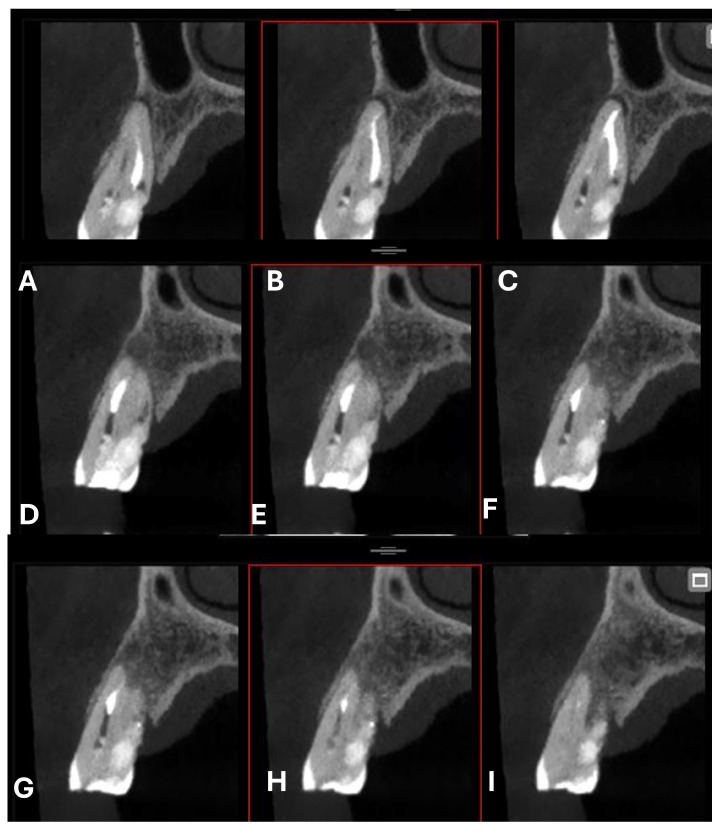
Cross-section CBCT images of tooth 1.4 after six months of healing. (**A**–**I**) Sequential scrolling of EPL healing.

**Figure 12:**
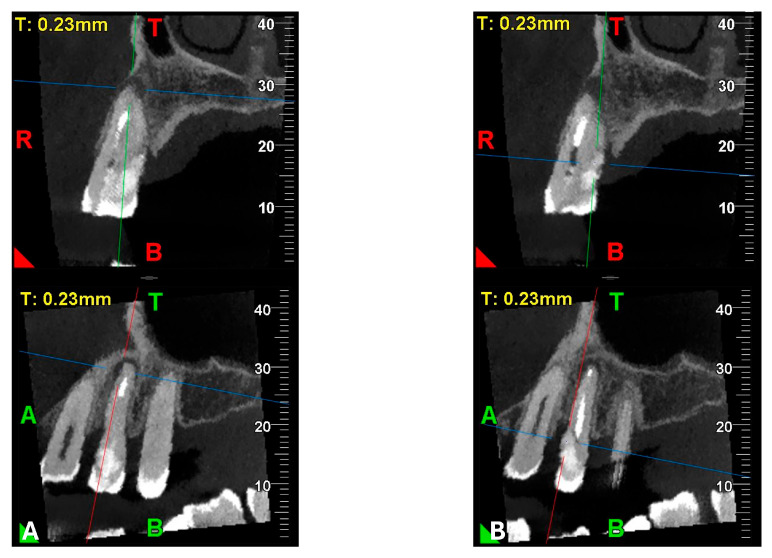
Pano-rex and cross-sectional CBCT images of tooth 1.4 after six months of healing. (**A**,**B**) Sequential scrolling of tooth 1.4 after six months of healing. The colored lines crossing the images represent the orthogonal planes (axial, coronal, and sagittal) in the multiplanar reconstruction. Their correct use and orientation allow for an accurate identification of the extent of the lesions in all spatial planes and a detailed analysis of the endodontic anatomy. The green and red letters are the coordinates of the image: A: anterior, R: right, B: bottom, T: Top.

**Figure 13:**
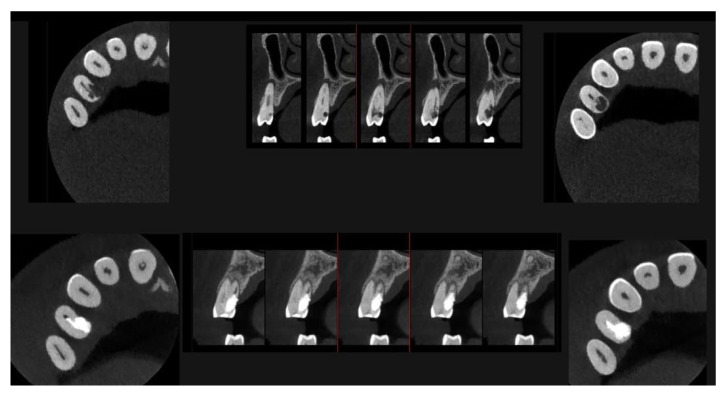
Pre-treatment and 6-month follow-up healing comparison of EPL.

**Figure 14:**
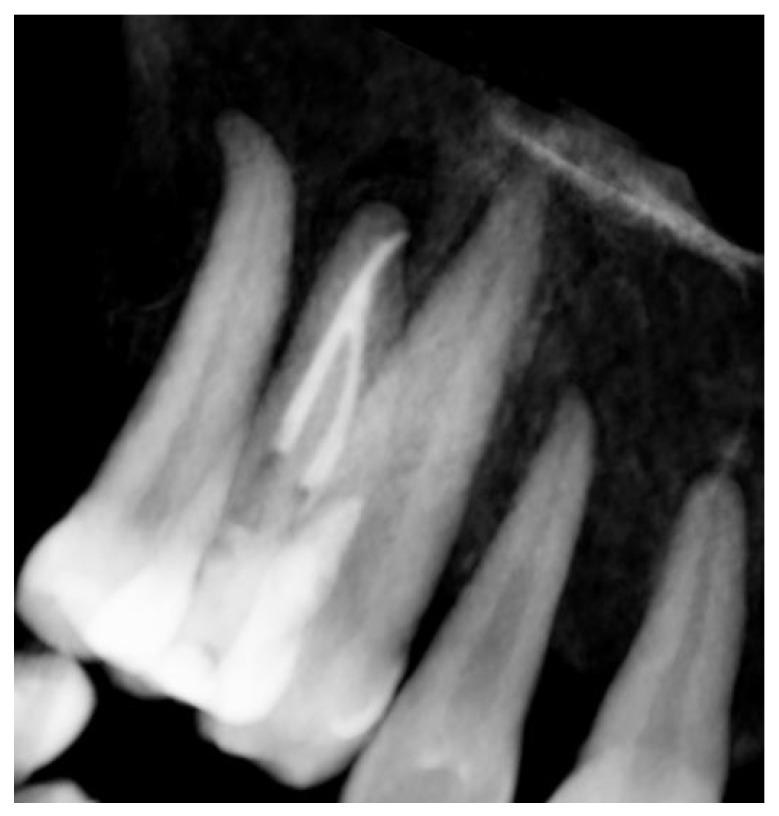
Rendering of CBCT images of tooth 1.4 after six months of healing.

An intermediate follow-up CBCT scan at 6 months was performed to accurately assess the response to treatment in light of both the extent of the lesion and the patient’s systemic comorbidities (Figure 10, Figure 11, Figure 12, Figure 13 and Figure 14).

**Figure 15:**
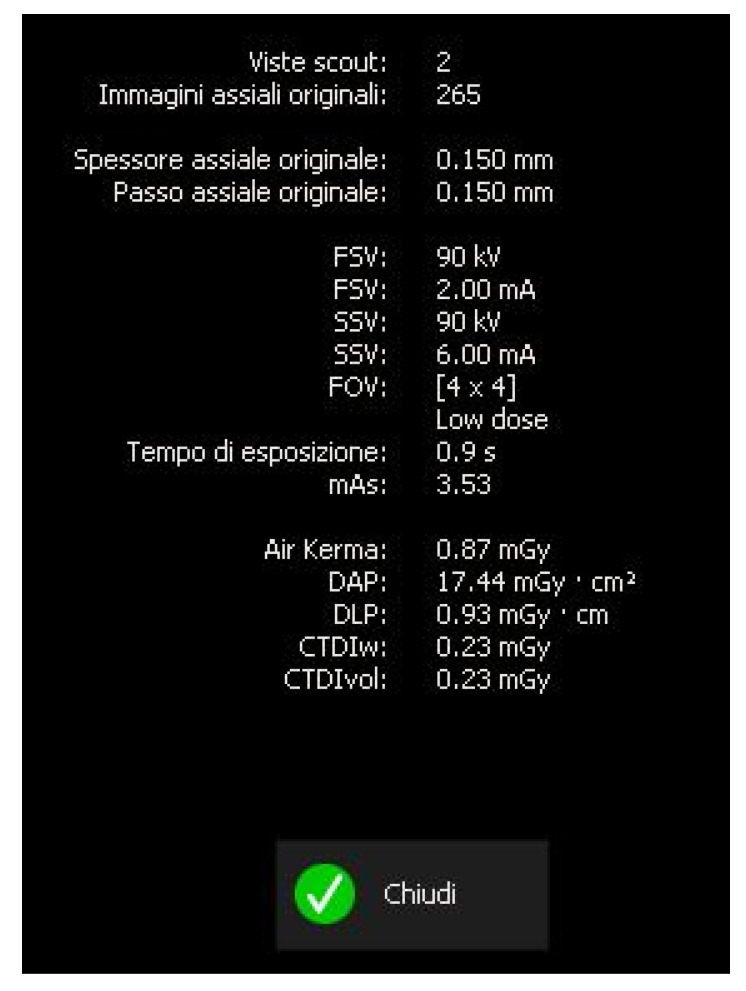
Exposure parameters of CBCT follow-up image.

The scan was carried out using a dedicated FOV, in this case 4 × 4 cm, with optimized exposure parameters tailored to the diagnostic question. Notably, the resulting dose-area product (DAP) was only 17 mGy·cm^2^—less than one-fourth of that generated by a standard panoramic radiograph (Figure 15)—while still providing diagnostic image quality suitable for both assessment and treatment planning, thus complying with the principles of optimization for radiological exposure. Attention to the use of low-dose CBCT, with clear diagnostic detail, constitutes a distinctive hallmark in the authors’ radiological approach.

**Figure 16:**
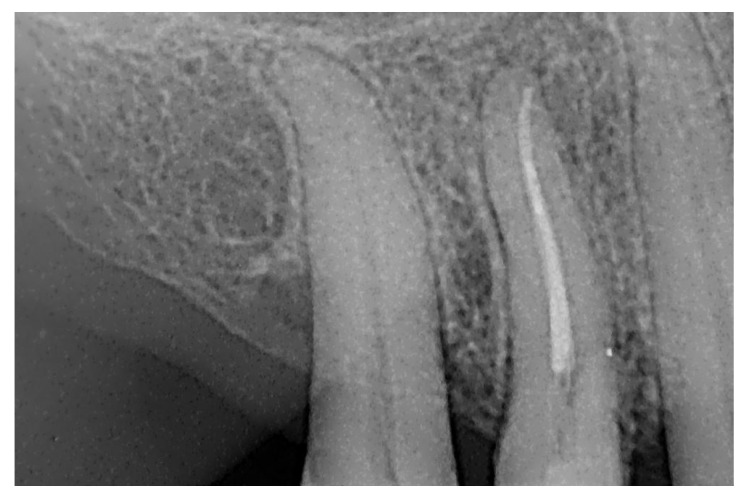
Intraoral radiography of tooth 1.4 at 12-month follow-up.

**Figure 17:**
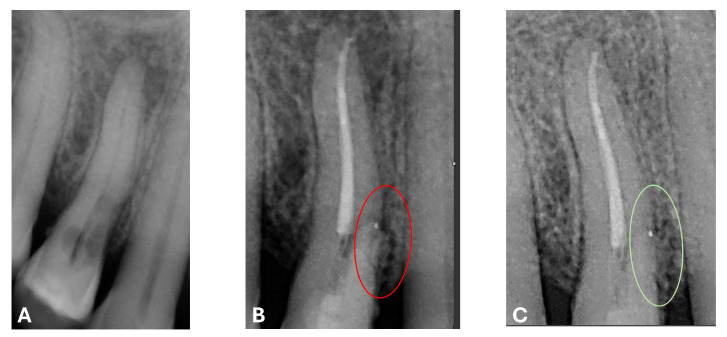
EPL before (**A**) and after treatment (red circle showing mesiocervical widening of the lamina dura space) (**B**) and at 12-month follow-up (**C**) with radiographic evidence of restoration of the lamina dura (green circle), periodontal ligament space and absence of PL.

At the 12-month radiographic follow-up, healing of the EPL was confirmed. Restoration of the lamina dura and the periodontal ligament adjacent to the treated site was observed (Figure 16 and Figure 17).

## 3. Discussion

When approaching the treatment of a patient with compromised systemic health, whether the intervention is endodontic, periodontal, or surgical, it is essential to carry out a thorough medical history to gain a comprehensive overview and identify all potential warning signs that influence clinical management and treatment planning.

Numerous systemic factors—including age, pregnancy, cardiovascular diseases, bleeding disorders, malignant neoplasms, chronic metabolic conditions such as diabetes, and transmissible infectious diseases such as acquired immunodeficiency syndrome (AIDS) and hepatitis—significantly and consistently affect the therapeutic approach to the involved tooth. These variables influence key aspects of clinical management, such as the anesthetic technique and pharmacological choices, the need for antibiotic prophylaxis, the indication for a conservative versus extraction-based approach, the selection between nonsurgical endodontic therapy (NSET) and surgical endodontic therapy (SET), as well as the possible temporary suspension of long-term medications.

Smoking has been significantly associated with delayed healing of periapical lesions following RCT, as reported in a recent study [26]. This trend appears to be more pronounced with increased exposure to smoking, with a higher risk observed in individuals smoking more than 20 cigarettes per day. Notably, in the referenced study, all patients were treated by endodontic specialists.

For these reasons, the endodontist must possess a solid understanding of the core principles of clinical medicine and the interactions between systemic conditions and both endodontic and oral pathologies, particularly when managing patients with complex medical histories [6].

In patients with a history of bisphosphonate therapy, endodontic treatment followed by restorative procedures is generally considered safe. However, a potential association has been reported between endodontic therapy and the development of bisphosphonate-related osteonecrosis of the jaw (BRONJ). For this reason, rubber dam placement must be performed with particular care to avoid trauma to the soft tissues, which could increase the risk of BRONJ. Additionally, BRONJ may arise following infections caused by the extrusion of debris through the apical foramen during root canal treatment. Consequently, the patient should perform a one-minute rinse with chlorhexidine prior to the procedure to reduce the oral bacterial load. Complete removal of carious tissue is indicated before initiating endodontic therapy [6].

Patients undergoing oncologic chemotherapy have an increased susceptibility to infections. Before initiating endodontic treatment in such individuals, hematological parameters (including white blood cell, inflammatory biomarkers and platelet counts and coagulation function) must be carefully evaluated. It is also important to assess the condition of the oral mucosa to identify any increased risks of bleeding or infection. To minimize the risk of infection during treatment, the patient should maintain proper oral hygiene, and the clinician must avoid trauma to the periodontal tissues, strictly adhering to aseptic protocols [6].

An additional study identifies how systemic risk factors and the patient’s immune response may influence the healing of endodontically treated teeth, emphasizing that the success of endodontic treatment does not solely depend on the quality of the procedure itself, but rather on the patient’s immunological and inflammatory response [2].

The patient in this case report was affected by endometrial cancer and was undergoing oncologic pharmacological suppression therapy. For this reason, the treatment of the EPL resulting from ECR needed to be as conservative as possible. It was therefore decided to proceed with RCT using a cold single-cone obturation technique, combined with the application of bioceramic cements in two formulations: sealer and putty.

The ability of calcium silicate-based materials to interact with surrounding tissues—eliciting different biological responses depending on the context of contact—forms the basis of their bioactivity and biocompatibility [27,28]. In the present case, the contact between the Biodentine^®^ and the periodontal tissue initiated a reparative process involving the formation of the mineralized tissue and likely the periodontal ligament, as demonstrated in various animal and in vivo studies [29,30].

The strong alkalinity of these materials appears to play a key role in activating alkaline phosphatase (ALP), thereby enhancing osteoblastic differentiation, inhibiting osteoclastic activity, and increasing the production of a mineralized matrix. Moreover, the biological behavior of resin-free bioceramic cements is superior to that of resin-containing formulations, showing significantly more favorable effects when in contact with periodontal ligament cells [31].

Given the case at hand, where the internal sealing of the resorption area is clearly limited—due to the defect’s wider external base on the root surface—the use of a material capable of promoting contact osteogenesis appears to yield better outcomes than a material that relies solely on perfectly sealing an endo-periodontal communication to initiate reparative processes aimed at *restitutio ad integrum*.

According to the therapeutic decision-making tree outlined in a recent expert consensus statement [10] published only a few months ago, the majority of teeth affected by EPL—regardless of the presence or absence of root damage—should initially undergo endodontic treatment, except in cases where extraction is indicated. Prior to considering periodontal surgical intervention, an appropriate period of clinical reassessment should be observed.

Proper root canal therapy is capable of effectively halting the spread of pathogens, managing pulpal infection, reducing pain and related symptoms, and promoting the regeneration of periodontal tissues. Moreover, in teeth affected by EPL with internal root resorption, endodontic treatment helps reduce the risk of root fractures or the need for extraction by removing infected debris and ensuring three-dimensional obturation of the root canals. A key determinant in the therapeutic outcome of EPL-affected teeth is the quality and adequacy of the endodontic therapy itself [4].

The European Society of Endodontology (ESE) has established specific criteria for the assessment of root canal treatment (RCT) outcomes, clearly defining the concepts of success and failure in endodontics [32].

In particular, primary orthograde endodontic therapy should be re-evaluated no earlier than one year after treatment. A favorable outcome (success) is defined by the presence of the following parameters:

- Absence of pain, swelling, or other clinical symptoms;
- Absence of sinus tract or exudate;
- Radiographic evidence of a normal periapical periodontal space, consistent with healing of the lesion.

The criteria for the successful treatment of EPL include the following: (1) a clinically appreciable improvement in symptoms and restoration of normal function of the affected tooth; (2) absence of recurrent abscesses or fistula formation; (3) reduction in deep periodontal pockets and improvement in alveolar bone height; and (4) control of pulpal and periapical inflammation, as evidenced by a clear resolution of periapical bone destruction visible in post-operative follow-up imaging [10].

In general, an extended follow-up period is preferable. The main radiographic changes following treatment tend to become evident after approximately three months. Therefore, it would be logical to propose a minimum observation period of three months. However, it is also essential to consider the possibility of residual chronic infections. For this reason, some authors recommend a minimum follow-up duration of four years [6].

At the time of treatment, the tooth presented as necrotic, with a combined apical and periodontal lesion clearly identifiable both clinically and radiographically.

In the present case report, a one-year follow-up is presented, demonstrating excellent progression of lesion healing, an increase in the height of the alveolar bone adjacent to the lamina dura, resolution of the pathological probing depth, recovery of the continuity of the periodontal ligament and lamina dura along the root surface and in correspondence with the previously affected area of the cervical resorption and lesion, and complete remission of odontogenic symptoms, restoring the functional integrity of the tooth [33]. Consequently, the efficacy of the treatment can be affirmed: through the use of bioceramic cements in two formulations and the implementation of a meticulously performed intraoperative endodontic technique, a successful therapeutic outcome was achieved, with the dental element remaining functional and in position within the arch.

A recent study published by Romeiro [1] highlighted that mechanical preparation and interim calcium hydroxide medication of the root canal may be comparably effective in both oncologic and non-oncologic patients, although this applies primarily to the management of apical infections.

In our case report, however, we document the effectiveness of a complete RCT not only in managing apical infection, but also in the healing of an EPL in an oncologic patient with significant systemic comorbidities, including diabetes mellitus. This approach goes beyond simple intracanal medication, demonstrating the therapeutic potential of endodontic intervention, even in complex clinical scenarios. To the best of the authors’ knowledge, no further studies on this topic have been reported.

We also introduce an evaluation of systemic inflammatory markers, in line with the recent literature linking them to reduced healing potential. We believe that the integration of these parameters, together with a comprehensive endodontic strategy, represents a clinically relevant approach worthy of further investigation, especially in medically compromised patients affected by multiple systemic conditions.

Thanks to the support and constant visual monitoring provided by the operating microscope during endodontic procedures, along with dedicated 3D radiologic planning, it was possible to customize and improve procedural accuracy [11], a crucial factor in fragile patients, in order to prevent, as much as possible, operative inaccuracies. The literature also reports that the precise placement of the rubber dam clamp is essential in these patients to avoid local trauma and bacterial contamination. Special care must also be taken to minimize the extrusion of debris beyond the apex, which, in selected patients, may lead to medication-related osteonecrosis of the jaw (MRONJ) and severe complications [34].

The preservation of the tooth through a single-visit procedure allowed for minimization of tissue trauma and microbial contamination associated with a second dental intervention, thus enabling the patient to focus on more pressing systemic health issues. In the present case, healing was not adversely affected by the patient’s severe systemic condition, in contrast to what is commonly reported in the literature [2,3].

To achieve an accurate diagnosis of the endo-periodontal lesion, according to Patel’s classification [21], the use of CBCT was essential. Unlike two-dimensional intraoral radiographs, CBCT provides greater sensitivity and specificity in identifying both periapical pathology and ECR.

In particular, for a category of patients whose current systemic conditions are especially severe or compromised, the use of CBCT, rather than the more commonly performed orthopantomography (OPT) as a first-level examination, ensures greater accuracy in detecting lesions that might otherwise go unnoticed with two-dimensional imaging until the onset of pain or significant lesion progression. For this reason, we agree with the conclusions of the study by Tempesta [34], which advocates the use of CBCT as a diagnostic tool prior to initiating therapies with antiresorptive or antiangiogenic drugs, in order to detect lesions, root fractures, or inadequate treatments.

Furthermore, in the management of medically fragile patients, the accurate identification of endodontic lesions through CBCT becomes especially relevant in candidates for structural cardiac surgery, patients with cardiac devices, or those affected by valvular heart diseases, as in the present case. In such individuals, CBCT, when performed with optimized exposure parameters to ensure the minimum effective dose compatible with adequate diagnostic information, allows not only for the assessment of pre-existing endodontic conditions, but also for the monitoring of treatment outcomes [22]. This makes CBCT a valuable tool for the early identification of potential recurrences or odontogenic infections that may pose a serious risk of systemic complications in these vulnerable patients. This statement is further supported by the 2017 french guidelines, endorsed by a broad consensus among various dental, medical and cardiology scientific societies [22,35].

### 3.1. Clinical Considerations and Practical Recommendations

In light of the challenges encountered in the management of the present clinical case, and based on the experience gained, the authors believe may be useful to provide a number of practical recommendations that could assist in the clinical approach to oncologic and/or systemically compromised patients presenting with complex endo-periodontal lesions:

- **Comprehensive Medical History and Systemic Evaluation Through a Multidisciplinary Approach:**
  A thorough collection of the patient’s medical history, ideally supported by consultation with clinical specialists (e.g., oncology and disciplines related to the patient’s comorbidities), is essential. This allows for a tailored and personalized therapeutic plan, including the timing and modality of endodontic treatment, based on the general systemic condition. Such an approach enhances the quality, precision, and predictability of clinical outcomes.
- **Dedicated and Integrated Use of Operative Microscopy and Radiographic Imaging, Particularly CBCT:**
  The appropriate and strategic use of CBCT provides superior diagnostic information in terms of both quality and quantity. It allows for the accurate spatial assessment of lesions, identification of radicular or subcrestal structural defects, and facilitates more precise planning of minimally invasive yet effective interventions. In complex clinical contexts, CBCT represents an essential tool for advanced and predictive diagnosis and treatment planning.
- **Use of Second-Generation Bioceramic Materials:**
  New-generation bioceramic cements offer an optimal solution for sealing and tissue regeneration in challenging clinical scenarios, owing to their high bioactivity, biocompatibility, and relatively short setting times. Their ability to maintain mechanical stability and harden even in acidic environments—typical of inflamed tissues—makes them particularly suitable for patients with compromised healing capacities, such as those who are immunosuppressed or undergoing oncologic treatments.
- **Future Research Directions:**
  In this vulnerable patient population, quality of life emerges as a central clinical priority, and future research should aim not only to identify the most effective treatment strategies, but also to go beyond the mere resolution of infection and the functional preservation of the dental element, encompassing a broader and sustainable contribution to the patient’s overall well-being and emotional resilience, especially within the context of complex and multidisciplinary care.

### 3.2. Limitations

This case report also presents certain limitations, most notably the small sample size. Although the single-visit protocol adopted in this case led to a significant improvement, both subjective and objective, in the initial condition, a larger patient cohort would be necessary to validate the effectiveness of the proposed approach. Understandably, this poses a challenge, given the complex systemic conditions of the patients who would need to be enrolled in such a study. Moreover, some authors recommend a longer follow-up period of up to four years in the event of residual chronic EPL. However, such long-term monitoring is not always feasible, particularly in patients affected by debilitating systemic conditions [6].

The limitations related to the recruitment of oncologic and immunocompromised patients, often presenting with complex systemic frailty, should not discourage the development of future multidisciplinary studies aimed at enriching the literature and identifying evidence-based, reproducible therapeutic strategies for endodontic management.

## 4. Conclusions

Comprehensive patient-centered evaluation is fundamental for the effective personalization of precision treatments [36]. Nevertheless, this crucial step is frequently overlooked in favor of technical procedures and specific clinical protocols.

In the case presented, the endodontic treatment proved to be both safe and effective, despite the patient’s complex systemic and pharmacological profile. The approach successfully addressed common complications reported in the literature, such as increased infection risk, chronic inflammation, and the reduced efficacy of root canal therapy in similar clinical contexts. Performing the procedure in a single session significantly reduced the time burden on the patient, an essential consideration in the care of individuals with systemic or oncological conditions.

The use of advanced technologies and two formulations of bioceramic materials played a key role, owing to their bioactivity and biocompatibility. The treatment not only resolved the odontogenic infection, but also alleviated symptoms and discomfort, contributing to an overall improvement in the patient’s quality of life. This outcome is particularly important in patients with limited therapeutic windows and complex health conditions.

Ultimately, this case highlights the importance of an integrated approach that considers the patient’s overall condition—beyond technical expertise and available technologies—as a means to optimize outcomes and improve care strategies. The favorable clinical results encourage further research and clinical exploration aimed at enhancing dental management in patients with complex systemic needs and limited prognostic timelines.

## Data Availability

Data Availability Statements are available upon request to the corresponding authors.

## Supplementary Materials

The following supporting information can be downloaded at: https://www.mdpi.com/article/10.3390/dj13090388/s1, Video S1: “Orthograde direct visualization of the root resorption site under operating microscope, followed by obturation with Biodentine”.

## Abbreviations

The following abbreviations are used in this manuscript:

EPL	endo-periodontal lesion
ECR	External cervical resorption
RCT	Root canal treatment
CBCT	Cone beam computed tomography
CRP	C-reactive protein
Hs-CRP	high-sensitivity C-reactive protein
IL-6	interleukin-6
IL-1β	interleukin-1 beta
TNF	tumor necrosis factor
MTA	Mineral Trioxide Aggregate
hPDLSCs	human periodontal ligament stem cells
IRR	invasive internal root resorption
IER	invasive external root resorption
ESE	European Society of Endodontology
CEJ	cemento-enamel junction
CVD	cardiovascular disease
HbA1c	glycated hemoglobin
PL	periapical lesion
FOV	field of view
NiTi	nickel-titanium
NaOCl	sodium hypoclorite
EDTA	ethylenediaminetetraacetic acid
DAP	dose-area product
AIDS	Acquired immunodeficiency syndrome
SET	surgical endodontic therapy
NSET	nonsurgical endodontic therapy
BRONJ	bisphosphonate-related osteonecrosis of the jaw
ALP	alkaline phosphatase
MRONJ	medication related osteonecrosis of the jaw
OPT	orthopantomography
